# Inositol pyrophosphate synthesis by diphosphoinositol pentakisphosphate kinase-1 is regulated by phosphatidylinositol(4,5)bisphosphate

**DOI:** 10.1042/BSR20171549

**Published:** 2018-03-16

**Authors:** Vasudha S. Nair, Chunfang Gu, Agnes K. Janoshazi, Henning J. Jessen, Huanchen Wang, Stephen B. Shears

**Affiliations:** 1 The Signal Transduction Laboratory, National Institute of Environmental Health Sciences, National Institutes of Health, 111 T.W. Alexander Drive, Research Triangle Park, North Carolina 27709, U.S.A.; 2 Institute of Organic Chemistry, Albert-Ludwigs University, Freiburg, Albertstr. 21, 79104 Freiburg, Germany

**Keywords:** compartmentalization, inositol pyrophosphates, inoitol, kinase, phosphatase, signalling

## Abstract

The 5-diphosphoinositol pentakisphosphate (5-InsP_7_) and bisdiphosphoinositol tetrakisphosphate (InsP_8_) are “energetic” inositol pyrophosphate signaling molecules that regulate bioenergetic homeostasis. Inositol pyrophosphate levels are regulated by diphosphoinositol pentakisphosphate kinases (PPIP5Ks); these are large modular proteins that host a kinase domain (which phosphorylates 5-InsP_7_ to InsP_8_), a phosphatase domain that catalyzes the reverse reaction, and a polyphosphoinositide-binding domain (PBD). Here, we describe new interactions between these three domains in the context of full-length human PPIP5K1. We determine that InsP_7_ kinase activity is dominant when PPIP5K1 is expressed in intact cells; in contrast, we found that InsP_8_ phosphatase activity prevails when the enzyme is isolated from its cellular environment. We approach a reconciliation of this disparity by showing that cellular InsP_8_ phosphatase activity is inhibited by C_8_-PtdIns(4,5)P_2_ (IC_50_ ~40 μM). We recapitulate this phosphatase inhibition with natural PtdIns(4,5)P_2_ that was incorporated into large unilamellar vesicles. Additionally, PtdIns(4,5)P_2_ increases net InsP_7_ kinase activity 5-fold. We demonstrate that PtdIns(4,5)P_2_ is not itself a phosphatase substrate; its inhibition of InsP_8_ phosphatase activity results from an unusual, functional overlap between the phosphatase domain and the PBD. Finally, we discuss the significance of PtdIns(4,5)P_2_ as a novel regulator of PPIP5K1, in relation to compartmentalization of InsP_7_/InsP_8_ signaling *in vivo*.

## Introduction

**Figure 1:**
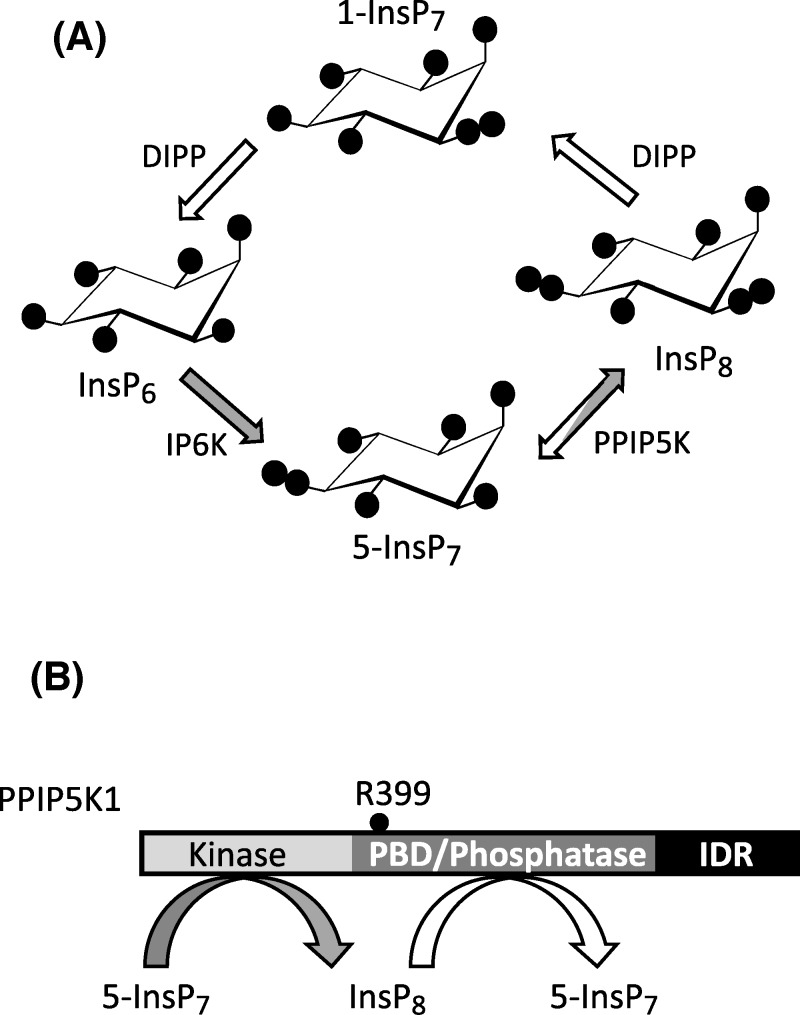
The contributions of PPIP5Ks to PP-InsP metabolism *in vivo* (**A**) A proposed cyclical pathway of PP-InsP turnover (see [1]). The arrows depicting the various reactions are colored white to denote phosphatase activity, and gray to describe kinase activity; DIPP, diphosphoinositol polyphosphate phosphatase; IP6K, inositol hexakisphosphate kinase. (**B**) Domain structure of PPIP5K1; the location of the catalytically essential R399 is indicated; PBD, polyphosphoinositide-binding domain; IDR, the noncatalytic, intrinsically disordered domain (see [37]).

Inositol pyrophosphates (PP-InsPs) are small, diffusible molecules with seven ( “InsP_7_” ) or eight ( “InsP_8_”) phosphates crammed around the six-carbon inositol ring (Figure 1A); the PP-InsPs act at the interface between cell-signaling and metabolic circuitry [1], a process that is essential to cellular and organismal homeostasis [2]. Thus, there is considerable interest in the enzymes that regulate the metabolism of the PP-InsPs and hence control their signaling activities. Among these enzymes are the diphosphoinositol pentakisphosphate kinases (PPIP5Ks). These are large, modular proteins; four domains have been recognized: a 5-InsP_7_ kinase activity, an InsP_8_ phosphatase, a polyphosphoinositide-binding domain (PBD), and a protein-scaffolding domain (Figure 1B) [1]. The existence within a single protein of opposing kinase and phosphatase activities—a “ futile” cycle—is a particularly specialized signaling nexus that is typically associated with multiple regulatory inputs [3,4]. In contrast, with regards to the PPIP5Ks, little is known of mechanisms by which their competing catalytic activities might be controlled. That is, we have limited insight into dynamic control over cellular InsP_8_ levels. Consequently, the recent demonstration that InsP_8_ is a suppressor of both glycolytic and mitochondrial [ATP] production [5] lacks regulatory context.

Mammals express both PPIP5K1 and PPIP5K2 [6–8]. These are predominantly cytoplasmic enzymes, although a certain amount of PPIP5K1 also associates with plasma membranes, at least in some mammalian cell types, such as NIH3T3 cells [9] and L6 myoblasts [10]. Moreover, the size of the plasma membrane pool increases when the phosphatidylinositol 3-kinase (PI3K) pathway is activated by either osmotic stress or growth factors [9,10]. It has been proposed that this stimulus-dependent translocation of human PPIP5K1 is driven by increased synthesis of PtdIns(3,4,5)P_3_, a ligand for the PBD [9]. It has been further shown that a R399A mutation within the PBD of PPIP5K1 elicits an approximately 10-fold reduction in affinity for PtdIns(3,4,5)P_3_ [9]. We were especially drawn to the latter observation, because R399 is also required for InsP_8_ phosphatase activity [11]. This suggests there is structural overlap between the phosphatase domain and the PBD, which has prompted us to now investigate if the binding of PtdIns(3,4,5)P_3_ might influence phosphatase activity.

Human PPIP5K1 has a molecular weight of 160 kDa; recombinant versions of such large proteins are inherently difficult to obtain, particularly when they are also labile—as are the PPIP5Ks [8]. In such cases, the general ease with which smaller, individual domains can be expressed in plentiful quantities is a boon for a reductionist approach to functional studies. Indeed, it was studies with recombinant PBD expressed in *Escherichia coli* that originally identified PtdIns(3,4,5)P_3_ as the preferred polyphosphoinositide ligand [9]. However, there is always a possibility that working with individual protein domains may sacrifice contextual breadth, a problem that is exacerbated when separate domains are functionally interrelated [12]. In other words, the functions of individual domains may be modified by other regions of the full-length protein. This concern prompted us to undertake the first characterization of the properties of the PBD in the context of the full-length protein. We determine that it is not PtdIns(3,4,5)P_3_, but PtdIns(4,5)P_2_ that is the preferred ligand *in vivo*. Our data lead us to redefine the functional significance of the interactions of polyphosphoinositides with PPIP5K1.

## Materials and methods

### *Drosophila* S3 cell culture

*Drosophila* S3 cells were maintained in Schneider’s complete medium, supplemented with 10% heat-inactivated fetal bovine serum (Gibco). As previously described [13], we prepared strains of S3 cells stably transformed with either the pMT/BioEase-DEST destination vector hosting full-length human PPIP5K1 (S3^PPIP5K1^ cells), or vector alone (S3^vector^). Another strain of S3 cells was transformed with vector hosting a R399A phosphatase-compromised PPIP5K1 mutant [11], which itself was prepared using the Q5 site-directed mutagenesis kit (New England Biolabs). The primers (mutagenic nucleotides underlined) were as follows: forward, TGCAATTATTGCTCATGGGGATCGTACTC; reverse, ATGACACAACGAAGTTCC. Gene expression was induced through the metallothionein promoter [13].

### Assay of [^3^H]inositol-labeled PP-InsPs in intact cells

To assay inositol phosphates, 6 × 10^6^ of S3^vector^ and S3^PPIP5K1^ cells were seeded in a 10-cm dish and cultured for 3 days in 10 ml of medium supplemented with 10 μCi/ml [^3^H]inositol (American Radiolabeled Chemicals) at which point both cultures were 70% confluent. Cells were acid-quenched, and the inositol phosphates were extracted and analyzed with a 3 × 250-mm CarboPac^TM^ PA200 HPLC column (ThermoFisher Scientific) as described in [11].

As previously described [10], rat myoblast L6 cells were cultured in 6 cm dishes and radiolabeled with [^3^H]inositol for 5 days, before transfer to 3 ml of a serum-free medium for 2 h prior to the addition of either 50 ng/ml platelet-derived growth factor (PDGF) or 100 nM insulin. Cells were then quenched, neutralized, and analyzed by HPLC [10].

### Preparation of large unilamellar vesicles (LUV)

Large unilamellar vesicles were prepared with the aid of an Avanti Mini Extruder using standard procedures [14]. Their composition is as follows (all components purchased from Avanti): 27% phosphatidylinositol (PtdIns); 26.5% phosphatidylethanolamine, 25% phosphatidylserine; 12% phosphatidylcholine; 3% sphingomyelin; 2% cholesterol; 4.5% 1-stearoyl-2-arachidonoyl-PtdIns(4,5)P_2_. In separate control vesicles, PtdIns(4,5)P_2_ was replaced with additional PtdIns.

### Purification and assay of PPIP5K1

All enzyme purification and assay procedures were performed in an anaerobic chamber (5% hydrogen, 95% nitrogen). After induction of PPIP5K1 expression, the recombinant protein was captured using avidin beads, as previously described [13]. Next, the beads were washed three times with 1 ml of wash buffer (50 mM HEPES, pH 8.0, 150 mM NaCl, 0.1% NP40, 1 mM EDTA, 2 mM DTT). Proteins were then eluted from the beads by two washes with 0.2 ml elution buffer (50 mM HEPES, pH 8.0, 150 mM NaCl, 1 mM EDTA, 2 mM DTT, 10 mM biotin). The concentration of the free PPIP5K1 was determined by SDS/PAGE and detection by silver staining, with densitometric analysis by comparison with standards of PPIP5K1^1-956^ [15]. Enzyme aliquots were stored at −80°C.

Phosphatase assays of PPIP5K1 were performed for 30–35 min at 37°C in 120 μl buffer (unless otherwise indicated) containing 20 mM HEPES (pH 7.2), 100 mM KCl, 0.6 mM MgCl_2_, plus either 1 μM chemically synthesized InsP_8_ [16], supplemented with trace amounts of [^3^H]InsP_8_ [17], or trace quantities of GroP[^3^H]InsP(4,5)P_2_ (deacylated Ptd[^3^H]Ins(4,5)P_2_; [18]). The quantity of enzyme added was 200 ng, unless otherwise stated. When polyphosphoinositides were also present, these were preincubated with enzyme for 1 h on ice before addition of [^3^H]InsP_8_. In assays that included LUVs, these were added at a final concentration of 8% (v/v). Reactions were quenched, neutralized, and analyzed by Partisphere SAX HPLC as previously described [11,18]. Representative HPLC chromatographs are shown for illustrative purposes. Time course experiments (see the Results and discussion section) demonstrated that, in our reaction conditions, reaction rates proceeded linearly for at least 30 min. We did not perform time courses in phosphatase assays for the PPIP5K1^R399A^ mutant, which exhibits negligible phosphatase activity; these assays were only implemented for 2 h.

In some other experiments, we examined if PPIP5K1 could dephosphorylate either C_8_-PtdIns(4,5)P_2_, or alternately, 1-stearoyl-2-arachidonoyl-PtdIns(4,5)P_2_ when it was presented to the enzyme in LUVs (see above). We recorded inorganic phosphate release using a sensitive microplate assay [19].

The 5-InsP_7_ kinase activities were measured at 37°C in 2 h incubations comprising 120 μl of assay buffer containing 1 mM Na_2_EDTA, 50 mM KCl, 20 mM HEPES (pH 7.2), 7 mM MgCl_2_, 5 mM ATP, 0.5 mg/ml BSA, trace amounts of 5-[^3^H]InsP_7_ [17] and, unless otherwise stated, 1 μM of chemically synthesized 5-InsP_7_ [20]. Reactions were quenched, neutralized, and analyzed by Partisphere SAX HPLC as previously described [11,18]. Each preparation of enzyme that was used for a kinase assay was prevalidated with a phosphatase assay to check for catalytic consistency. In preliminary experiments, we found that 2 h was the earliest time-point at which we could accurately assay [^3^H]InsP_8_ accumulation. Thus, we did not conduct a time course analysis.

### Confocal immunofluorescence

The S3^PPIP5K1^ and S3^vector^ cells were seeded on MatTek 35 mm dishes in complete Schneider’s medium containing 10% FBS (Gibco). After 24 h induction of vector or PPIP5K1, cells were fixed with 3.7% paraformaldehyde in phosphate-buffered saline (PBS) for 15 min. After two rinses in PBS, the dishes were incubated in PBS containing 0.1 M glycine and 0.1% TritonX-100 for 6 min. After two more rinses with PBS, fixed cells were blocked in PBS containing 3.5% BSA for 30 min and processed for immunofluorescence by incubation for 1 h with the anti-PPIP5K1 antibody (Sigma-Aldrich) diluted 1:300 in PBS-BSA. The dishes were washed in PBS twice before addition of secondary antibody (Alexa Fluor FITC mouse IgG) for 40 min. After three washes in PBS, dishes were stained with Hoechst 33358 for 5 min. Cells were washed twice and images were acquired with either Zeiss LSM780 or LSM880 confocal microscopes (Carl Zeiss MicroImaging) equipped with a EC Plan-Neofluar 40 x/1.30 DIC M27 oil and Apochromat 40 x/1.2 W Korr FCS M27 water objectives. Excitation wavelengths were 488 nm for FITC (emission between 500 and 540 nm) and 405 nm for the Hoechst (emission between 420 and 480 nm). Both were operated by Zen software (version 2012; Zeiss). Images were processed and analyzed with ImageJ (National Institutes of Health) or Photoshop (Adobe).

## Results and discussion

### Exogenous expression of PPIP5K1 in *Drosophila* S3 cells elevates levels of 1-InsP_7_ and InsP_8_

**Figure 2:**
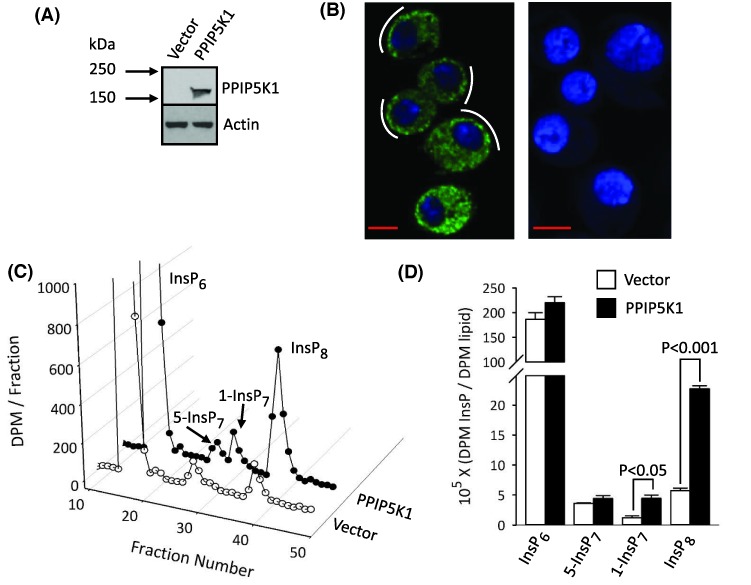
The impact of heterologous expression of PPIP5K1 upon PP-InsP turnover in *Drosophila* S3 cells (**A**) Western analysis of PPIP5K1 expression in S3^vector^ and S3^PPIP5K1^ cells. (**B**) Confocal immunofluorescence analysis of PPIP5K1 distribution and Hoechst staining in S3^PPIP5K1^ cells (left panel) or S3^vector^ cells (right panel); white curved lines highlight a tendency for the PPIP5K1 to be more concentrated in punctae near the plasma membrane; scale bars = 5 μm. (**C**) HPLC chromatograph of InsP_6_ and PP-InsPs in [^3^H]inositol-labeled S3^vector^ and S3^PPIP5K1^ cells. (**D**) Cellular levels of InsP_6_ and PP-InsPs, normalized to levels of [^3^H]inositol-lipids, from three independent experiments similar to that described in panel C.

We first established a *Drosophila* S3 cell line as an inducible expression system for PPIP5K1. These cells are an inexpensive and robust model that reduces the experimental variability inherent in transient expression, while limiting long-term cellular consequences of stable expression [13,21]. The degree of induction of PPIP5K1 expression in S3^PPIP5K1^ cells was monitored by Western analysis (Figure 2A) and by confocal immunofluorescence, which showed that PPIP5K1 was distributed throughout the cell, although some more concentrated punctae were proximal to the plasma membrane (Figure 2B). A similar subcellular distribution has been noted previously, for PPIP5K1 that was heterologously expressed in rat L6 myoblasts [10].

We next checked functionality of PPIP5K1 in S3 cells by monitoring its influence upon the PP-InsP profile. In fact, as far as we are aware, PP-InsP turnover has not previously been demonstrated in any *Drosophila* cell-type (see, for example, [22]), although the fly genome encodes both IP6K and a single PPIP5K. Here, by HPLC analysis of [^3^H]inositol-labeled S3^vector^ cells, we found that both 5-InsP_7_ and InsP_8_ were present at approximately equal levels (Figure 2C,D). It is striking that the S3^PPIP5K1^ cells contained 4-fold higher levels of InsP_8_ following induction of PPIP5K1 (Figure 2C,D).

In previous work with both HEK293 and HCT116 cells, we could not detect significant amounts of 1-InsP_7_, even following PPIP5K1 overexpression [5,23]. We now demonstrate that 1-InsP_7_ is also barely detectable in S3^vector^ cells (Figure 1C,D). In contrast, levels of 1-InsP_7_ in S3^PPIP5K1^ cells are similar to those of 5-InsP_7_ (Figure 2C,D). According to the pathway of PP-InsP turnover described by Figure 1A, 1-InsP_7_ could accumulate as a consequence of increased cyclical flux through InsP_8_. However, there may also be increased PPIP5K-mediated phosphorylation of InsP_6_ to 1-InsP_7_ [7,11]. In either case, our observations demonstrate that, in the intact S3-cell environment, the poise of the competing kinase and phosphatase activities of PPIP5K1 (Figure 1B) are such that the kinase is favored.

### The kinase and phosphatase activities of purified recombinant PPIP5K1

**Figure 3:**
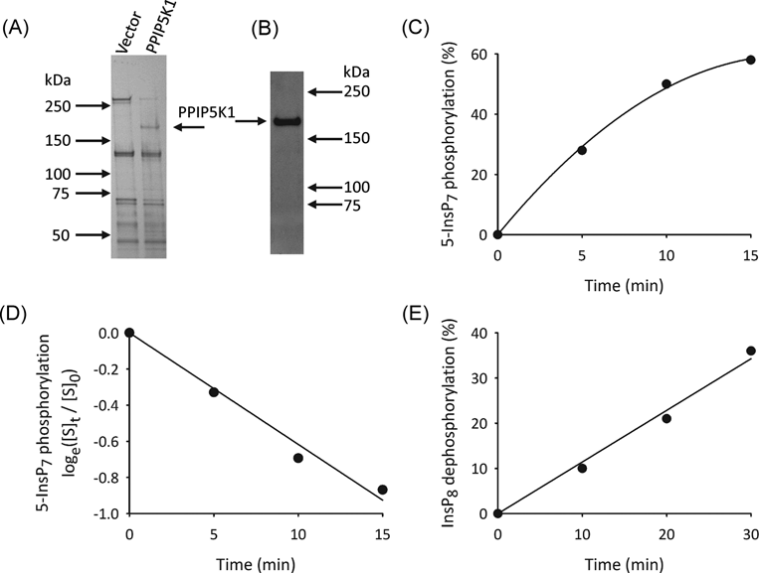
Analysis of recombinant PPIP5K1 (**A**) Silver-stained, SDS/PAGE analysis of recombinant PPIP5K1 purified from S3^PPIP5K1^ cells, compared with S3^vector^ cells. (**B**) Western analysis of recombinant PPIP5K1. (**C**) Representative time course of 5-InsP_7_ phosphorylation by 30 ng PPIP5K1 and just trace amounts of [^3^H]-labeled substrate (i.e under first-order conditions [24]). (**D**) Natural logarithmic transformation of the data in panel C, where [S]_0_ = [^3^H]substrate at time zero, and [S]*_t_* = [^3^H]substrate at time *t*, as indicated. (**E**) Time course of InsP_8_ dephosphorylation by 220 ng recombinant PPIP5K1, determined by HPLC.

Recombinant PPIP5K1 was purified from S3^PPIP5K1^ cell lysates, using avidin-coated beads to bind the N-terminal, biotinylated BioEase tag; PPIP5K1 was liberated from the beads using biotin. The integrity of the free protein was confirmed by SDS/PAGE (Figure 3A; molecular weight = 190 kDa) and by Western analysis (Figure 3B). We initially verified the active 5-InsP_7_ kinase activity of PPIP5K1 under first-order conditions i.e., in assays with just trace amounts of 5-[^3^H]InsP_7_ (Figure 3C). A natural logarithmic transformation of these data (Figure 3D) provides the first-order rate constant (0.07 min^−1^) which, being proportional to *V*_max_, is considered to faithfully define the activity of an enzyme toward its substrate (see [24]).

**Figure 4:**
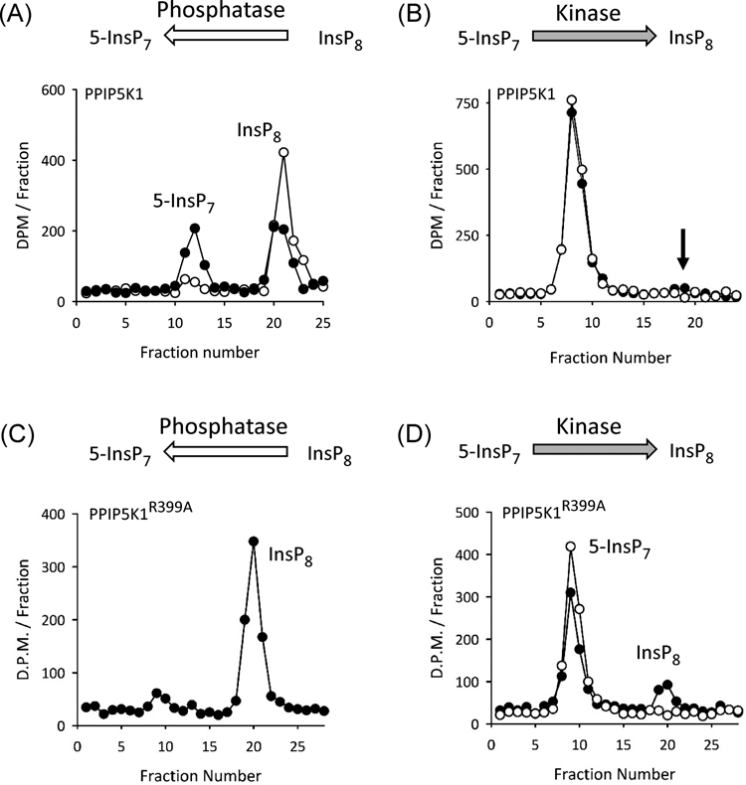
InsP_8_ phosphatase and 5-InsP_7_ kinase activities of recombinant PPIP5K1 (**A**) HPLC analysis of InsP_8_ phosphatase activity of 220 ng recombinant PPIP5K1 incubated for zero min (open circles) and 30 min (closed circles). (**B**) HPLC analysis of 5-InsP_7_ kinase activity of 346 ng recombinant PPIP5K1 incubated for zero min (open circles) and 2 h (closed circles); the arrow marks the expected elution position of InsP_8_. (**C**) Representative InsP_8_ phosphatase activity of 570 ng recombinant PPIP5K1^R399A^ incubated for 30 min. (**D**) Representative 5-InsP_7_ kinase activity of 346 ng recombinant PPIP5K1^R399A^ incubated for zero min (open circles) or 2 h (closed circles).

We obtained biologically relevant enzyme activities by incubation of PPIP5K1 with concentrations of substrates (i.e. 1 μM) that are close to those present in intact cells. Active InsP_8_ phosphatase activity was reliably recorded under these conditions (Figures 3E and 4A). The average reaction rate is 7 ± 1 nmol/mg protein/min (*n*=11). In contrast, the net phosphorylation of 1 μM 5-InsP_7_ was quite small (Figure 4B), presumably because of the competing activity of the InsP_8_ phosphatase. We established in preliminary time course experiments that 2 h assays were the minimum duration required to detect InsP_8_ accumulation. One example is shown in Figure 4B (the arrow indicates the HPLC elution position of InsP_8_, which amounts to 2.3% of total [^3^H] present in the assay i.e., 0.7% / ng protein).

Our proposal that the active phosphatase activity of PPIP5K1 prevents significant accumulation of InsP_8_ was tested by assaying net 5-InsP_7_ kinase activity of the PPIP5K1^R399A^ phosphatase-compromised mutant (Figure 4C,D). In these experiments, to derive a direct comparison with the wild-type enzyme (Figure 4B), we incubated a similar amount of PPIP5K1^R399A^ for 2 h, and determined that InsP_8_ accumulation exceeded 20% of total [^3^H] in the assay (Figure 4D). In three similar experiments, the average amount of InsP_8_ accumulation was 6.9 ± 0.01% / ng protein.

Clearly, the capacity of wild-type PPIP5K1 to synthesize InsP_8_ *in vitro* (Figure 4B) does not recapitulate the enzyme’s ability to elevate InsP_8_ accumulation in intact cells (Figure 2C,D). Thus, we propose that the inherently dominant kinase activity *in vivo* must be the result of regulatory factors that are not present in our assay *in vitro*.

### The effects of insulin and growth factors upon PP-InsP turnover in intact mammalian cells

**Figure 5:**
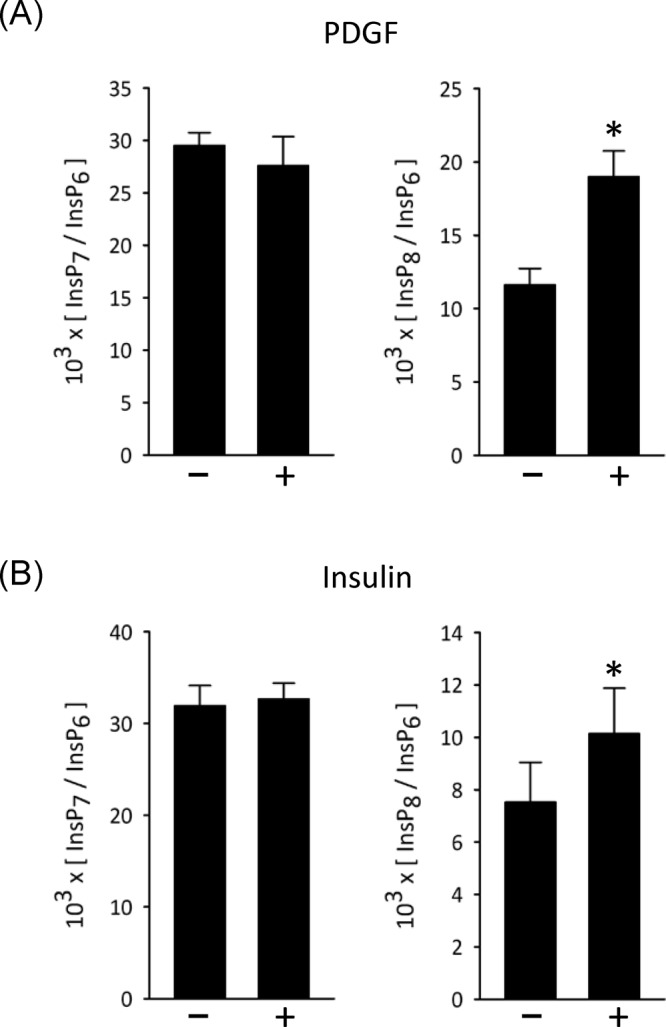
Effects of PDGF and insulin upon InsP_7_ and InsP_8_ levels in L6 myoblasts [^3^H]inositol-labeled L6 myoblasts were incubated for 30 min with either (**A**) 50 ng/ml platelet-derived growth factor or (**B**) 100 nM insulin. Cells were then quenched, neutralized, and analyzed by HPLC. Vertical bars depict means and SE for 3–5 experiments; *P<0.05. Quantitatively similar results were obtained with 5 and 10 min agonist exposure times.

To understand how net 5-InsP_7_ kinase activity of PPIP5K1 may be enhanced *in vivo* (see above), we followed up previous work, in which we stimulated mammalian cells with either growth factors or hyperosmotic stress; in these earlier experiments, PI3K was activated and PPIP5K1 translocated to the plasma membrane [10]. This is a situation in which binding of polyphosphoinositides to the PBD of PPIP5K1 [9] may be increased. Furthermore, other previously published data indicate that cell activation with growth factors can modestly enhance InsP_8_ accumulation: 45–70% increases were observed following either 30 min treatment of DDT1MF-2 cells with 100 ng/ml EGF [25] or 5 min treatment of NIH3T3 cells with 50 ng/ml PDGF [10]. We have now re-examined this phenomenon using a cell-type (rat L6 myoblasts) in which insulin-mediated recruitment of PPIP5K1 by plasma membranes is especially prominent, and also sustained [10]. We found that either PDGF or insulin elicits statistically significant increases in InsP_8_ levels in L6 cells (Figure 5A,B). The degree of these effects is again quite modest (35–64%), but specific nevertheless; neither PDGF nor insulin affected 5-InsP_7_ levels (Figure 5A,B). These data bring attention to the possibility that the receptor-dependent elevation of [InsP_8_] in intact cells could reflect regulation of PPIP5K1 catalytic activities by binding of polyphosphoinositides.

### The phosphatase activity of PPIP5K is inhibited by polyphosphoinositides

**Figure 6:**
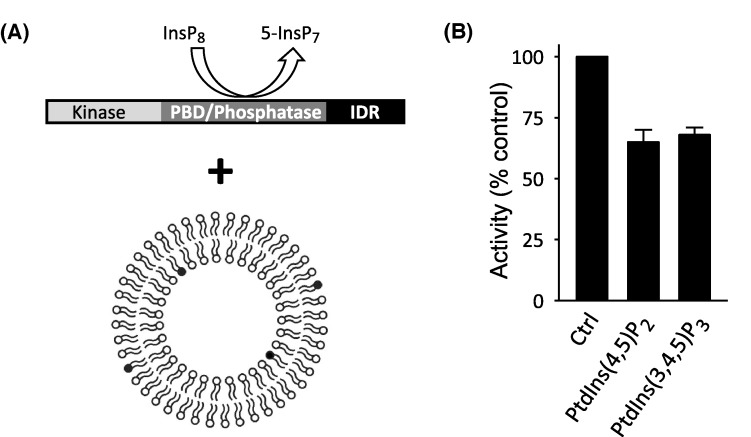
Effects upon InsP_8_ phosphatase activity of large unilamellar vesicles containing either PtdIns(4,5)P_2_ or PtdIns(3,4,5)P_3_ (**A**) Graphic depicting the assay; the incubation of LUVs with full-length PPIP5K1. The dark gray shading of four phospholipid head groups indicates that the concentration of the test polyphosphoinositide is 4.5%. (**B**) InsP_8_ phosphatase activities estimated from assays containing 100–150 ng of recombinant PPIP5K1, incubated for 35 min with LUVs containing 4.5% PtdIns(3,4,5)P_3_ or PtdIns(4,5)P_2_. Vertical bars depict means and SE for three experiments.

To pursue possible interactions of polyphosphoinositides with PPIP5K1, we next investigated if the enzyme’s InsP_8_ phosphatase activity is affected upon incubation of the enzyme with PtdIns(4,5)P_2_ in a physiologically relevant format i.e., as a minor constituent of an overall anionic surface of a large unilamellar vesicle (LUV) (Figure 6A). We found that LUVs containing 4.5% PtdIns(3,4,5)P_3_ inhibited InsP_8_ phosphatase activity by approximately 30% compared with LUVs lacking any polyphosphoinositide (Figure 6B). To judge the biological relevance of this effect, it should be noted that the plasma membranes of human erythrocytes contain 1.5% PtdIns(4,5)P_2_ [26], but since the majority lies in the inner leaflet [27], the biologically effective level is 3%. Other mammalian cell types may contain higher amounts of PtdIns(4,5)P_2_ [28,29]. Furthermore, clustering of PtdIns(4,5)P_2_ molecules [27,30] sustains biologically active pools with locally elevated levels; a 3-fold increase above average PtdIns(4,5)P_2_ levels was recorded in a single molecule, super-resolution imaging study [30]. Another study described plasma membrane microdomains in which the local PtdIns(4,5)P_2_ concentration approached 80% [31]. These considerations argue that there is physiological relevance to our observation that InsP_8_ phosphatase activity is inhibited by LUVs containing 4.5% PtdIns(4,5)P_2_ (Figure 6B).

We investigated if InsP_8_ phosphatase activity is inhibited by PtdIns(4,5)P_2_ by virtue of the lipid being an alternative, competing substrate. For this experiment, we determined if inorganic P_i_ accumulated in assays in which PPIP5K1 (200 ng) was incubated with LUVs containing PtdIns(4,5)P_2_. To gain sufficient assay sensitivity, the incubation time was increased from 30 min to 3 h, the quantity of LUVs in the assay was increased 8% to 30% (v/v), and the concentration of PtdIns(4,5)P_2_ in the LUVs was elevated from 4.5% to 30% (i.e. a total of 1.8 nmol/assay). We performed control experiments that lacked enzyme in order to determine the assay variability: 0.06 ± 0.05 nmol P_i_. This value was not exceeded in parallel assays that contained PPIP5K1: 0.05 ± 0.01 nmol P_i_. We conclude that PtdIns(4,5)P_2_ is not a substrate of PPIP5K1, consistent with previous data showing a tight specificity for the 1-β-phosphate of InsP_8_ [13].

**Figure 7:**
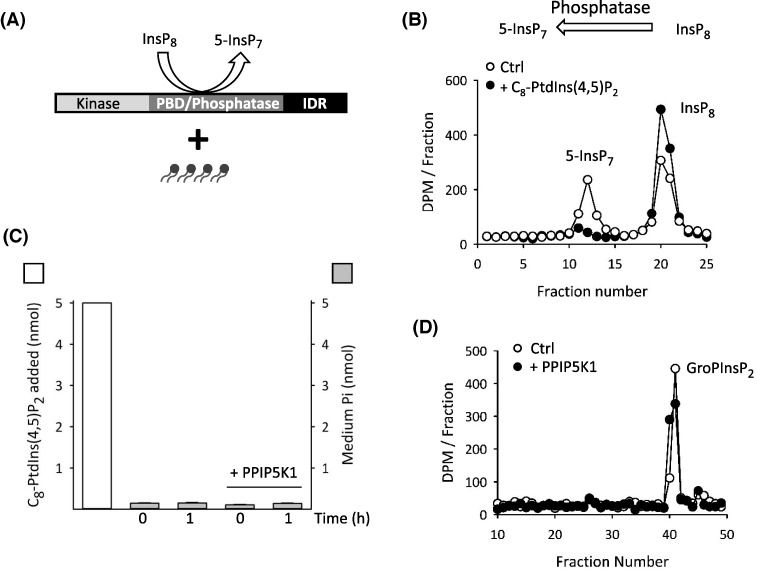
Inhibition of InsP_8_ phosphatase activity by a soluble, C_8_ analogue of PtdIns(4,5)P_2_ (**A**) Graphic depicting the assay; the incubation of C_8_ analogue of PtdIns(4,5)P_2_ with full-length PPIP5K1. (**B**) HPLC analysis of InsP_8_ phosphatase activity of 200 ng recombinant PPIP5K1 incubated in the absence (open circles) or presence (closed circles) of 50 μM C_8_-PtdIns(4,5)P_2_. (**C**) Gray bars describe P_i_ in assay medium (means ± SD; *n*=3), after incubation in 100 μl assays of 50 μM C_8_-PtdIns(4,5)P_2_ for either 0 or 1 h, either in the presence or absence of PPIP5K1 (200 ng). For comparison, the amount of added C_8_-PtdIns(4,5)P_2_ is also indicated (white bar). (**D**) HPLC analysis showing GroP[^3^H]Ins(4,5)P_2_ (the soluble, deacylated version of Ptd[^3^H]Ins(4,5)P_2_) is not dephosphorylated in either zero min (open circles) or 30 min (closed circles) incubations with 634 ng PPIP5K.

We next found that LUVs containing PtdIns(3,4,5)P_3_ were as effective as PtdIns(4,5)P_2_ at inhibiting PPIP5K1-catalyzed InsP_8_ phosphatase activity (Figure 6A,B). This finding seems quantitatively inconsistent with an earlier report of a 6-fold higher affinity of PtdIns(3,4,5)P_3_ for the isolated PBD of PPIP5K1, as compared with PtdIns(4,5)P_2_ [9]. We therefore investigated if the relative affinities of polyphosphoinositides for the PBD are altered in the context of the full-length protein. We are unable to record polyphosphoinositide-binding parameters directly, because the techniques for doing so (such as surface plasmon resonance) require much larger quantities of full-length PPIP5K1 than we can currently purify. Instead, we decided to recruit polyphosphoinositide-mediated inhibition of InsP_8_ phosphatase activity (Figure 6B) as a reporter assay for ligand affinity. To directly quantify the data obtained, we now used soluble C_8_-analogues of polyphosphoinositides (Figure 7A). The maximum concentrations of the lipid analogues used in these experiments (50 μM; Figure 7B) are at least 20-fold below their expected critical micelle concentrations [32]. We found that 50 μM of C_8_-PtdIns(4,5)P_2_ inhibited InsP_8_ dephosphorylation by approximately 60% (Figure 7A,B).

In control experiments in which we assayed inorganic P_i_, we did not detect any PPIP5K1-mediated dephosphorylation of C_8_-PtdIns(4,5)P_2_ (Figure 7C). Furthermore, we did not observe any PPIP5K1-mediated dephosphorylation of trace amounts of soluble, deacylated Ptd[^3^H]Ins(4,5)P_2_ (Figure 7D). Bearing in mind that such first-order assay conditions faithfully define the activity of an enzyme toward its substrate (see above and [24]), these data (Figure 7D) confirm our conclusion stated above that PPIP5K1 does not hydrolyze the inositol phosphate headgroup of PtdIns(4,5)P_2_.

**Figure 8:**
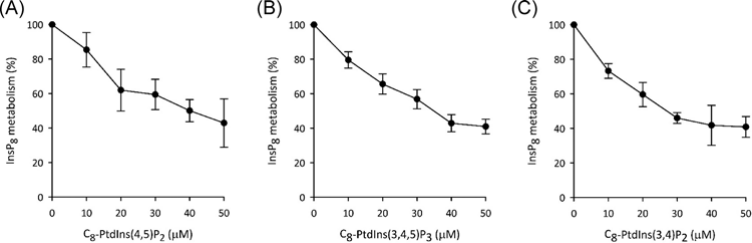
Dose–response analysis of the inhibition of InsP_8_ phosphatase activity by recombinant PPIP5K1 by polyphosphoinositides (**A**), (**B**), and (**C**) are dose-dependent effects upon InsP_8_ phosphatase activity of C_8_-PtdIns(4,5)P_2_, C_8_-PtdIns(3,4,5)P_3_, and C_8_-PtdIns(3,4)P_2_ respectively; data are means ± SEM from four experiments.

Dose–response studies (Figure 8A) generated an approximate IC_50_ value of 40 μM for inhibition of the phosphatase activity by C_8_-PtdIns(4,5)P_2_. Previous work with the isolated PBD showed that there are two other polyphosphoinositide ligands, PtdIns(3,4,5)P_3_ and PtdIns(3,4)P_2_ [7]. We found that C_8_-versions of these lipids also inhibited the InsP_8_ phosphatase activity of PPIP5K1. The approximate IC_50_ values for PtdIns(3,4,5)P_3_ and PtdIns(3,4)P_2_ (35 and 25 μM respectively; Figure 8B,C) are very similar to the value obtained for PtdIns(4,5)P_2_ (Figure 8A). Nevertheless, PtdIns(4,5)P_2_ is by far the most abundant of these polyphosphoinositides *in vivo*, at effective concentrations of approximately 5 mM ([33]); we conclude that PtdIns(4,5)P_2_ is the ligand that is the most physiological relevant for regulating InsP_8_ metabolism.

**Figure 9:**
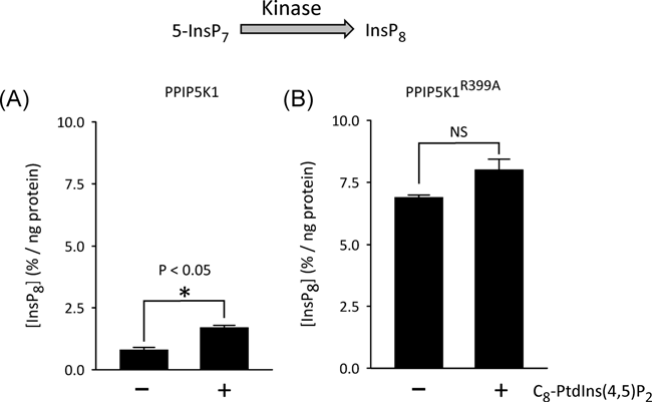
The effects of C_8_-PtdIns(4,5)P_2_ upon net 5-InsP_7_ phosphorylation by wild-type PPIP5K1 and phosphatase-dead PPIP5K1^R399A^ Bars depict net accumulation of InsP_8_ (means ± SE; *n*=3) after either (**A**) wild-type PPIP5K1 or, (**B**) phosphatase-dead PPIP5K1^R399A^ was incubated for 2 h with 1 μM 5-InsP_7_ in either the presence or absence of 50 μM C_8_-PtdIns(4,5)P_2_; *P<0.05; NS = not significant.

Finally we utilised full-length PPIP5K1 to determine whether inhibition of the InsP_8_ 1-phosphatase by PtdIns(4,5)P_2_ would result in elevated InsP_8_ accumulation during 5-InsP_7_ phosphorylation. For these experiments we used the more experimentally tractable C_8_-PtdIns(4,5)P_2_, rather than PtdIns(4,5)P_2_ in LUVs. We found that the degree of accumulation of InsP_8_ was enhanced 2.1-fold by the addition of 50 μM C_8_-PtdIns(4,5)P_2_ (Figure 9A). The fact that PtdIns(4,5)P_2_ increases apparent kinase activity indicates that inhibition of phosphatase activity by the lipid is not a consequence of a nonspecific, detrimental action upon enzyme structure.

Furthermore, C_8_-PtdIns(4,5)P_2_ did not have a statistically significant effect upon inherent kinase activity, which we determined using phosphatase-dead PPIP5K1^R399A^ (Figure 9B). Thus, we conclude that it is through inhibition of the phosphatase domain that C_8_-PtdIns(4,5)P_2_ (and presumably PtdIns(4,5)P_2_ *in vivo*) indirectly increases InsP_8_ accumulation by the kinase domain of wild-type PPIP5K1.

## Concluding comments

The present study ascribes new functional significance to the PBD of PPIP5K1: PtdIns(4,5)P_2_-mediated inhibition of InsP_8_ phosphatase activity, thereby promoting higher net InsP_7_ kinase activity. This is a significant observation because it allows us to appreciate how the cell can regulate the levels—i.e., the signaling strength—of a subplasmalemmal pool of InsP_8_.

A portion of total cellular PPIP5K1 may associate with the plasma membrane by default (Figure 2B) [9]. There is also an increased translocation of PPIP5K1 to the plasma membrane following stimulation of cells with growth factors or during hyperosmotic stress [10]. In both cases, InsP_8_ levels are also elevated (Figure 5 and see [10]). However, growth factors elicit a more modest increase in [InsP_8_] (35–64%; Figure 5) than that typically associated with hyperosmotic stress (5-fold; [10]). In the latter case, additional regulatory mechanisms presumably act upon the PPIP5Ks, but as yet their nature is unknown [34]. Nevertheless, it is worth noting that our estimates of total cellular InsP_8_ will underestimate the true extent of any changes to a localized, subplasmalemmal pool. To pursue this phenomenon in future work, it will be necessary to develop intracellular imaging technology for InsP_8_.

In our present study using full-length PPIP5K1, we found that PtdIns(4,5)P_2_, PtdIns(3,4)P_2_, and PtdIns(3,4,5)P_3_ each inhibits InsP_8_ phosphatase activity with approximately equal efficacy (Figures 6 and 8). This observation contrasts quantitatively with a previous demonstration that PtdIns(3,4,5)P_3_ has a 6-fold higher affinity for the isolated PBD than the other two lipids [9]. Conformational changes may explain how the relative ligand affinities of PBD might be modified by its incorporation into full-length PPIP5K1. In any case, we conclude that it is vital to use the full-length protein to derive physiologically relevant data on PPIP5K1 regulation *in vitro*.

While the present study is focused on discerning which polyphosphoinositide ligand might regulate InsP_8_ synthesis *in vivo*, our new data also lead us to reassess a conclusion from a previous study [9] that PtdIns(3,4,5)P_3_-binding to PPIP5K1 can directly drive recruitment of PPIP5K1 to the plasma membrane. This now seems a less viable primary recruitment mechanism, in view of our new conclusion that PtdIns(4,5)P_2_ is the preferred ligand. Perhaps it would be a productive direction for future research to investigate if other stimulus-dependent translocation mechanisms assist in plasma membrane recruitment of PPIP5K1. For example, insulin-mediated transfer to the plasma membrane of AKT is assisted by enhancing its association with the Arp2/3-mediated actin network [35]. Intriguingly, a yeast orthologue of PPIP5K1 has functional interactions with the Arp2/3 complex [36].

## Abbreviations

LUV: large unilamellar vesicles
PDB: polyphosphoinositide-binding domain
